# The effect of a reminder diary on risk factors in patients with chronic hypertension attending a clinic at a hospital in Johannesburg, South Africa

**DOI:** 10.4102/phcfm.v5i1.493

**Published:** 2013-07-30

**Authors:** Janine Webber, Aimee Stewart, Piet Becker

**Affiliations:** 1 Department of Physiotherapy, University of the Witwatersrand, South Africa; 2 Medical Research Council, Pretoria, South Africa

## Abstract

**Background:**

Poor adherence to lifestyle interventions and medication-taking is problematic, and there is some evidence that a diary may be useful in facilitating patients’ adherence to lifestyle modification in chronic disease.

**Objectives:**

To compare changes in blood pressure, waist–hip ratio, body mass index, blood levels and exercise capacity between two experimental groups and one control group (CG) after a six month intervention, and at a further three month follow up.

**Method:**

This was a longitudinal randomised control trial. All three groups underwent usual treatment. In addition, Experimental group one (EG1) received the diary as well as a once-a-month telephone call and Experimental group two (EG2) received only a once-a-month telephone call. Changes in measurements were established using an ANCOVA. The significance of the study was set at *p* = 0.05.

**Results:**

The added intervention of the diary had no direct effect on blood pressure change greater than that achieved by the appropriate medication. All three groups showed a clinically significant drop in both systolic and diastolic blood pressure to accepted norms. There were marginal differences in EG1 for waist–hip ratios (*p* = 0.06) at six months. There were significant low density lipoprotein (LDL) reductions in both EG1 and EG2 at nine months compared with the CG (*p* = 0.02) Walking distances improved minimally in both EG1 and EG2.

**Conclusion:**

The diary and telephone interventions showed some positive trends toward improvements in risk factors of patients with chronic hypertension.

## Abstrait

**L'effet d'un calendrier de rappels sur les facteurs de risque chez des patients souffrant d'hypertension chronique et traités dans un centre médical d'un hôpital de Johannesburg, en Afrique du Sud**

**Présentation:**

Le non respect des interventions relatives au mode de vie et de la prise de médicaments s'avère problématique, et il existe des preuves indiquant qu'un calendrier pourrait s'avérer utile pour faciliter le respect des changements de mode de vie par les patients souffrant de maladie chronique.

**Objectifs:**

Comparer les changements de pression artérielle, du rapport taille/hanches, de l'indice de masse corporelle, des taux sanguins et de la capacité d'exercice entre deux groupes expérimentaux et un groupe de contrôle (GC) après une intervention de six mois, puis trois mois après.

**Méthode:**

Cette étude consistait en un essai contrôlé randomisé et aléatoire. Les trois groupes ont suivi le traitement habituel. De plus, le Groupe expérimental 1 (GE1) s'est vu remettre le calendrier, et ses membres ont été contactés par téléphone une fois par mois; le Groupe expérimental 2 (GE2) a uniquement été contacté par téléphone une fois par mois. Les changements observés dans les mesures ont été établis par une analyse de la covariance. La signification statistique de l’étude a été fixée à *p* = 0.05.

**Résultats:**

L'intervention supplémentaire que constituait le calendrier n'a pas eu un effet direct sur le changement de pression artérielle supérieur à celui découlant d'un traitement médical approprié. Les trois groupes ont enregistré une baisse cliniquement significative de la pression artérielle systolique et diastolique à des niveaux acceptables. On a pu observer des différences marginales au sein du GE1 pour les rapports taille/hanches (*p* = 0.06) à six mois. On a pu observer des réductions significatives des lipoprotéines de faible densité (LDL) au sein du GE1 et du GE2 à neuf mois par rapport au CG (*p* = 0.02). La marche sur une certaine distance s’était très légèrement améliorée au sein du GE1 et du GE2.e

**Conclusion:**

Les interventions que constituent le calendrier et les appels téléphoniques ont montré qu'elles permettaient de réduire les facteurs de risque chez les patients souffrant d'hypertension chronique.

## Introduction

### Significance of work

This study sought to establish the effect of the use of a reminder diary to assist patients with hypertension in adhering to a healthy lifestyle. The results showed some improvements but illustrated yet again the difficulties in getting adherence to lifestyle changes from patients who are from poor socio-economic areas.

### Problem statement

Despite the large number of studies on patient adherence to lifestyle modification in chronic diseases such as hypertension, poor adherence to these modifications persists.^1, 2^ Patient adherence should be more than just adherence to medication prescription; it should include keeping clinic appointments, participating in regular exercise and adherence to recommended dietary and other lifestyle changes.^3^

The South African guidelines for the management of hypertension are rarely followed well in South African public hospitals.^4^ There are few or no interventions designed specifically to reduce cardiovascular risk factors by attempting to modify patients’ lifestyles.^4, 5, 6, 7, 8^ Most patients who utilise the public health care sector in South Africa are from poor socio-economic groups, are poorly educated, have poor knowledge and management of hypertension and do not follow a prudent lifestyle.^7, 9, 10, 11^

There are many reasons for non-adherence to preventive and therapeutic lifestyle recommendations amongst patients at high risk of cardiovascular disease. Patients’ main reasons for not adhering to lifestyle recommendations are unwillingness, difficulty in adhering to diets that are different from the rest of the family, the cost of prudent diets, the lack of time for exercise and the presence of other illnesses.^12^ There are very few interventions that demonstrate effective long-term adherence to medications and behaviour change. Therefore, new strategies are needed in order to identify successful methods to help patients adopt and maintain healthy lifestyle practices.^13, 14, 15^

Reminder diaries may be an intervention that improves adherence to lifestyle modification,^16, 17^ as they are visible reminders that can be reviewed by health care professionals during clinic visits and may motivate patients. Arrigo et al. showed that, in patients one year post-cardiac rehabilitation, using a diary and three-monthly group education sessions almost doubled patients’ adherence to exercise compared with patients who did not receive the diary or group sessions.^18^ This simple intervention may thus have the potential to improve adherence to lifestyle modifications in patients with hypertension in a public hospital.

The aim of this study was therefore to determine whether a reminder diary containing information designed to promote a healthy lifestyle in patients with chronic hypertension led to improvements in blood pressure control and cardiovascular risk factor modification in patients who attend a hypertension clinic at a public hospital.

## Research method and design

### Study design

A three-armed longitudinal randomised control trial was used to test the effectiveness of a reminder diary. All groups received the standard treatment at the hospital, namely, a once-a-month supply of medication and a once-in-three-monthly consultation with a medical officer at the clinic that also included measurement of weight and a glucose test. In addition, Experimental group one (EG1) was given the diary and had a once-a-month telephone call and Experimental group two (EG2) received a once-a-month telephone call. The control group (CG) received only the standard treatment at the clinic.

### Participants

Patients at a hypertension clinic at a regional hospital in Johannesburg, South Africa were sampled consecutively and were invited to participate in the study. On providing informed consent, they were randomised using computer-generated block randomisation and concealed allocation into three groups by a therapist not involved in the study.

Patients were included in the study if they were aged 40 years – 65 years; had been diagnosed with chronic hypertension (defined as having a blood pressure greater than 140/90 for more than 24 hours^19^); and were attending the hypertension clinic for the first time. Patients were excluded from the study if they had a previous history of severe and prevailing illness or disability or if they were on medication other than diabetic and antihypertensive drugs.

In order to measure a blood pressure change of 4 mmHg – 6 mmHg (SD = 4) at a significance level of *p* = 0.05, a sample size of 30 per group was required.^7^ A 15% dropout and 15% non-adherence rate were included in the sample size calculation. Accordingly, the initial sample size consisted of 90 participants, divided into 30 control participants and 60 experimental participants.

### Ethical considerations

Ethical approval was obtained from the Human Research Ethics Committee of the University of the Witwatersrand (Protocol number: M080521). Permission was granted for the study to be performed at the hospital and the participants signed informed consent before being included in the study. All participants continued to receive their usual treatment at the hypertension clinic for the duration of the study.

### Procedure

Demographic data (sex, race, educational level, annual income and cardiovascular risk factors) were collected at the start of the study by questionnaire.

The measurements discussed below were taken at baseline, after the 24-week intervention and after a further 12 weeks of no intervention. The first author took all the measurements and was blinded to group allocation and the results of the previous tests.

#### Blood pressure (primary outcome measurement) and exercise capacity

Exercise capacity was measured using the six-minute walk test.^19, 20^ Participants began the procedure by having their resting blood pressure (BP) tested, sitting with the left and right arm resting at shoulder height, after they had been sitting for 10 minutes. A calibrated digital sphygmomanometer was used to measure the participant's BP and heart rate at rest. A 20 m walkway was marked out with beacons on the floor of the hypertension clinic. The participants were instructed to walk as fast as possible between the beacons for six minutes. If they needed to rest, benches were available next to the measured 20 m strip. Every 30 seconds, the researcher said, ‘You are doing well, keep it up’. Immediately after the test, participants had their BP and heart rate tested on the left arm only and in the sitting position.^20^ The normal precautions for exercise testing^21^ were taken. After the test, the distance walked and the Rating of Perceived Exertion during walking were measured.^20^

#### Waist and hip circumference measurements

The participants were asked to remove all clothing around the waist (except very light underwear) and to loosen tight clothing. They were then asked to stand next to a mirror with gridlines, feet slightly apart, attempting to distribute their weight evenly on both feet. The assessor stood to the side of the participant and held the measuring tape firmly around the participant's waist at a level halfway between the lower rib margin and the iliac crest – just above the umbilicus^22^ – making sure that the tape was horizontal with the gridlines. The participants were asked to breathe in and out and the measurement was taken when the participants had breathed out. This prevented any inaccurate measurement due to the participants contracting their abdominal muscles or holding their breath. The measurement was recorded to the nearest millimetre.

The preparation and procedure for hip circumference measurement was as for waist circumference, except for the fact that the participants removed clothing around the hips and the measurement was taken around the hips at the level of the greatest circumference around the buttocks.

#### Weight measurement

A calibrated digital scale was used. The participants were asked to remove all clothing, except for light underwear, and to step onto the scale with feet slightly apart, distributing their weight as evenly as possible. The final reading on the scale was recorded.

#### Height measurement

A weighted string was used to ensure that the tape measure was vertical against a wall. The participants were asked to stand relaxed with their back against the tape measure on the wall and their height was recorded to the nearest millimetre.

#### Blood measurements

Bloods for glucose, cholesterol, and both High-density lipoprotein (HDL) and Low-density lipoprotein (LDL) and triglycerides were taken in the hospital blood room. The usual laboratory procedures at the hospital were followed.

#### Intervention

The reminder diary was drawn up in consultation with the staff of the hypertension clinic and a perusal of the literature.^16, 17^ The diary consisted of. simple basic information on hypertension with a new fact presented for reading on each consecutive day; suggestions for specific progressive exercises to do each day which were explained during the monthly telephone calls;^23^ reminders on what to eat and what not to eat (informed by the dietician in the clinic according to the Gauteng Department of Health healthy eating plan^24^); and a question on whether medication had been taken for that day. Participants ticked the appropriate column when the activity was completed. The dates when the therapist called the participant to follow up on the progress made (once a month) were also recorded in the diary. The diaries were available in English, Afrikaans, Sotho and Zulu. Any participant who had difficulty reading was encouraged to involve a member of their family in reading the diary. The diary was presented to EG1 by the research assistant who was a therapist and who explained carefully to the participants how to use the diary.

A second research assistant (therapist) telephoned EG1 and EG2 using the standardised script described below (see Box 1).

**BOX 1:** Standardised script used for telephone calls.

Greeting How are you doing? How is the medication, diet and exercise programme going? Are you managing to complete and adhere to the daily activities? Are you doing the daily exercises? If not, is there a problem? Are you taking the advice on eating? If not, is there a problem? Are you reading the tips everyday? If not, is there a problem? Do you have any questions about the medication, diet and exercise programme? Statement of encouragement Statement that if the participant needs to ask any questions, they may phone her (the research assistant). End phone call

*Source*: Standardised script devised for study.

### Statistical analysis

The three study groups were compared at baseline with respect to continuous parameters using a one-way analysis of variance (ANOVA) and with respect to discrete parameters using Pearson's Chi-square test. For the change in each of the outcome measurements, namely, BP, waist–hip ratio, weight and body mass index, glucose levels, and exercise capacity from baseline to six and nine months respectively, the control and intervention groups were compared using an analysis of covariance (ANCOVA) with the baseline value as covariate. Following a significant result in ANCOVA, specific differences amongst study groups were established from pair-wise comparisons using Fisher's least significant differences (LSD) approach. Testing was performed at the 0.05 level of significance.

## Results

**FIGURE 1:**
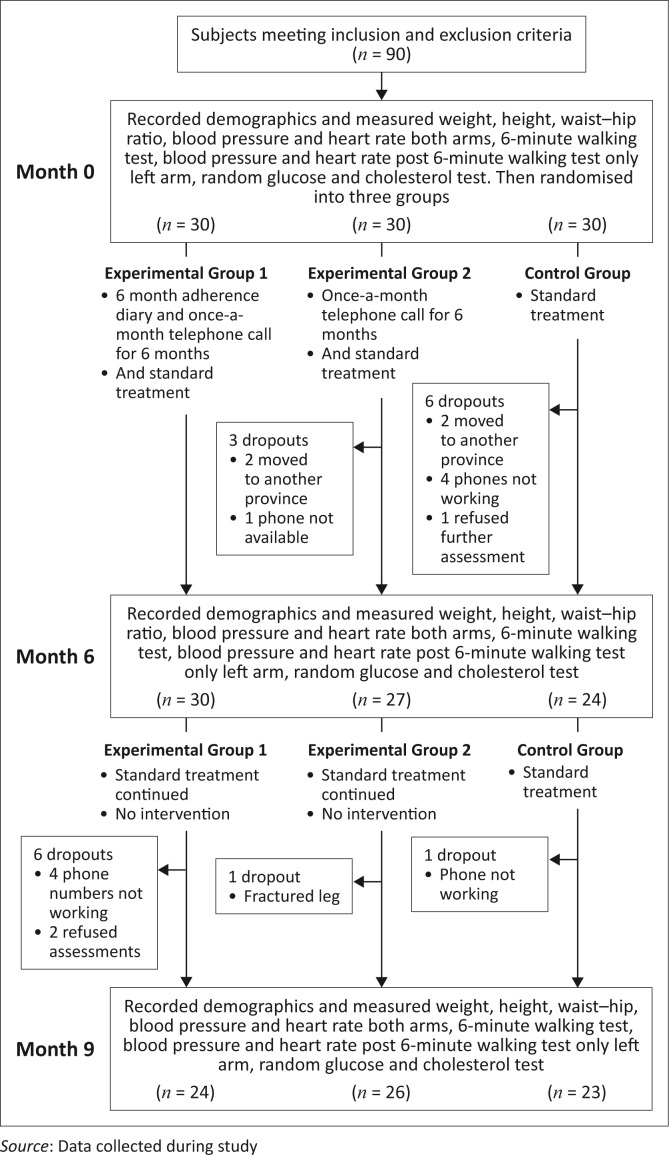
Flow of participants through study.

Figure 1 demonstrates the progression of participants through the study.

**FIGURE 2:**
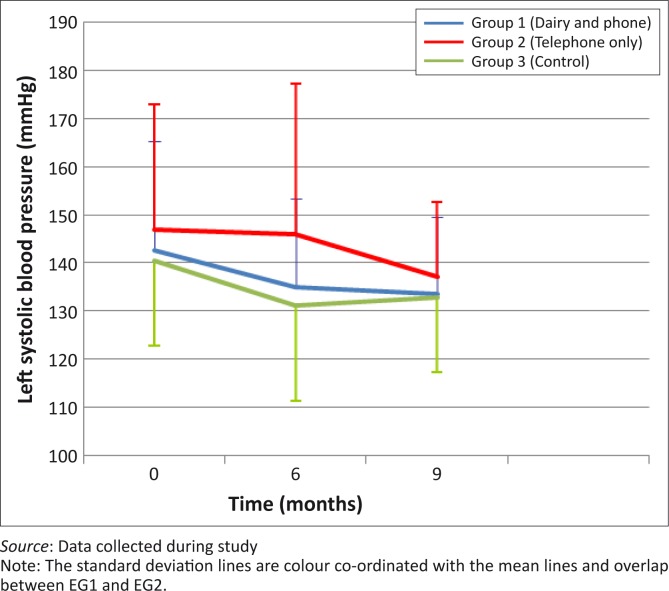
Mean left systolic blood pressure of groups over 9-month period.

**FIGURE 3:**
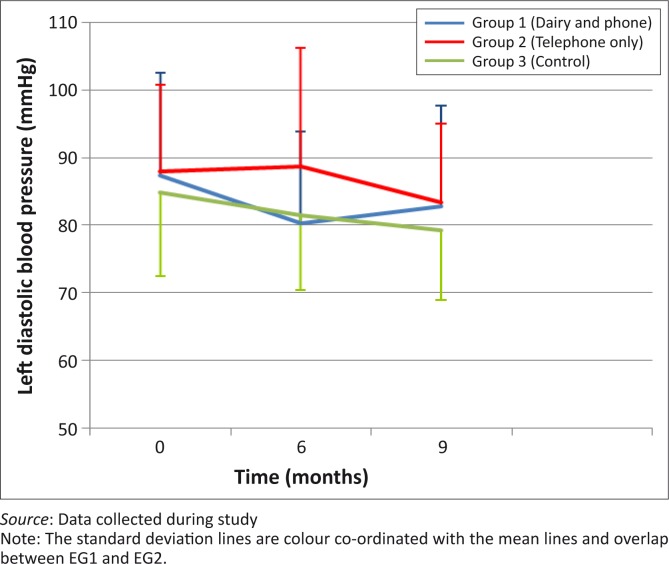
Mean left diastolic blood pressure of groups over 9-month period.

**FIGURE 4:**
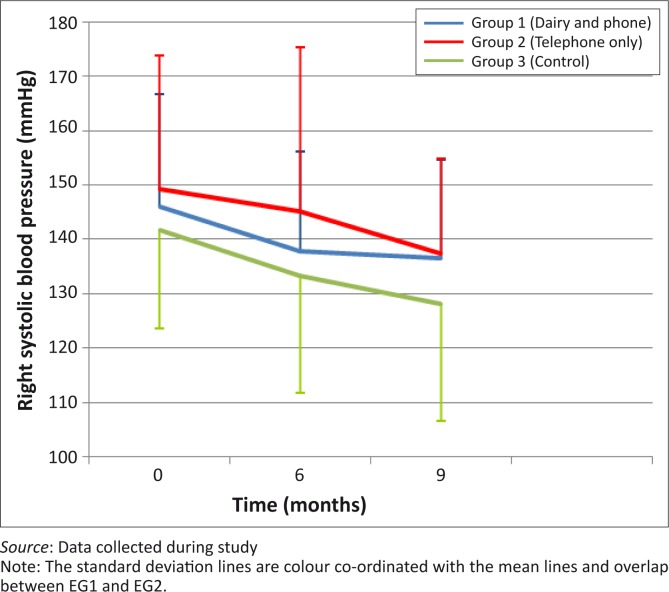
Mean right systolic blood pressure of groups over 9-month period.

**FIGURE 5:**
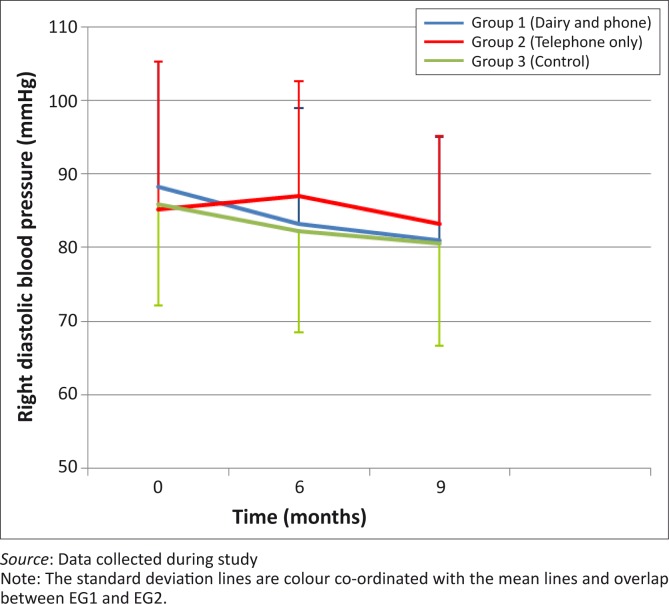
Mean right diastolic blood pressure of groups over 9-month period.

There were no statistically-significant differences in the decreases in left systolic BP between month 0 and month 6 (*p* = 0.34) and between month 0 and month 9 (*p* = 0.83) between the three groups (Figure 2). Figure 3 shows that left diastolic BP decreased in all groups between month 0 and month 6, with the greatest decrease shown in EG1, but with no significant between-group differences. All groups showed a decrease in right systolic BP between month 0 and month 6 as well as between month 0 and month 9, but without statistically-significant differences between the groups (Figure 4). Decreases in right diastolic BP were noted in EG1 and the CG between month 0 and month 6 (Figure 5).

Table 1 presents between-group comparisons with respect to the change from baseline in weight, BMI and waist–hip ratio at six and nine months. There was a marginal reduction in the waist–hip ratio in EG1 (*p* = 0.06). LDL was significantly reduced in both EG1 and EG2 compared with the CG at nine months (*p* = 0.02) (Table 2).

**TABLE 1:** Comparisons amongst groups with respect to change from baseline in weight, BMI and waist–hip ratio at 6 and 9 months.

Characteristics	Comparisons at 6 months				Comparisons at 9 months			
		
	EG1 m(SD)*	EG2 m(SD)	CG m(SD)	*p*-Val (*p*)	EG1 m(SD)	EG2 m(SD)	CG m(SD)	*p-*Val (*p*)
Weight (kg)	2.08 (6.12)	0.83 (2.75)	0.13 (4.61)	0.27	2.29 (7.03)	1.25 (2.89)	-0.02 (5.41)	0.32
BMI (kg/h^2^)	0.80 (2.38)	0.32 (1.09)	0.30 (1.78)	0.25	0.87 (2.69)	0.49 (1.14)	-0.02 (2.12)	0.30
Waist/Hip	-0.01 (0.04)	0.01 (0.05)	0.01 (0.04)	0.06	-0.01 (0.04)	0.00 (0.03)	0.00 (0.03)	0.35

*Source*: Data collected during study

* Note: m(SD) mean and standard deviation; EG1, Experimental group one; EG2, Experimental group two; BMI, Body Mass Index.

**TABLE 2:** Comparisons amongst groups with respect to change from baseline in blood levels at 6 months and 9 months.

Characteristics	Comparison at 6 months				Comparison at 9 months			
		
	EG1 m(SD)**	EG2 m(SD)	CG m(SD)	*p*-Val (*p*)	EG1 m(SD)	EG2 m(SD)	CG m(SD)	*p*-Val (*p*)
Glucose (mmol/l)	-0.41 (2.02)	-0.39 (3.27)	-0.15 (1.12)	0.71	-0.88 (2.49)	0.25 (2.85)	0.30 (1.42)	0.15
Cholesterol (mmol/l)	-0.10 (0.67)	-0.37 (1.09)	0.10 (0.94)	0.19	-0.39 (0.88)	-0.37 (0.87)	0.05 (0.99)	0.36
Triglycerides (mmol/l)	0.02 (0.36)	-0.16 (0.63)	0.24 (0.59)	0.11	-0.07 (0.58)	-0.12 (0.57)	-0.11 (0.46)	0.67
HDL (mmol/l)	0.26 (1.56)	-0.01 (0.16)	-0.01 (0.12)	0.51	0.03 (0.21)	0.00 (0.14)	0.01 (0.14)	0.68
LDL (mmol/l)	-0.01 (0.65)	-0.30 (0.95)	0.22 (0.99)	0.07	-0.39 (0.83)	-0.33 (0.84)	0.09 (0.85)	0.03 0.02* (2&3) 0.02* (1&3)

*Source*: Data collected during study

* Note: Pairwise *t*-tests revealing significant differences between two groups. m(SD), mean and standard deviation; EG1, Experimental group one; EG2 Experimental group two.

There were no statistically-significant differences in the changes in distance walked between month 0 and month 6 (*p* = 0.7) and between month 0 and month 9 (*p* = 0.69) between the three groups (Table 3).

**TABLE 3:** Comparisons amongst groups with respect to change from baseline in exercise capacity at 6 and 9 months.

Exercise capacity	Comparison at 6 months				Comparison at 9 months			
		
	EG1 m(SD)*	EG2 m(SD)	CG m(SD)	*p*-Val (*p*)	EG1 m(SD)	EG2 m(SD)	CG m(SD)	*p*-Val (*p*)
Distance (m)	-1.17 (73.40)	14.57 (44.51)	-2.69 (106.15)	0.70	19.29 (44.71)	12.36 (41.51)	1.81 (63.13)	0.69

*Source*: Data collected during study

* Note: Pairwise *t*-tests revealing significant differences between two groups. m(SD), mean and standard deviation; EG1, Experimental group one; EG2 Experimental group two.

The baseline characteristics of the sample are presented in Table 4 and show that the majority of patients in this study were female, black, either unemployed or on pensions, and had poor educational levels, although the educational levels in EG1 were higher than the other two groups (*p* = 0.04). Most of the sample did not exercise and had a variety of cardiovascular risk factors as shown in Table 5.

**TABLE 4:** Baseline characteristics of participants (*n* = 90; 30 participants per group).

Characteristics	Groups at baseline			*p*-value
		
	EG 1 *n*(%)	EG 2 *n*(%)	CG 3 *n*(%)	
**Sex**				0.41
Male	7 (23.33)	6 (20.00)	11 (36.67)	-
Female	23 (76.67)	24 (80.00)	19 (63.33)	-
**Race**				0.69
White	4 (13.33)	2 (6.67)	3 (10.00)	-
Black	16 (53.33)	18 (60.00)	18 (60.00)	-
Indian/Coloured	10 (33.33)	10 (33.33)	9 (30.00)	-
**Educational Level**				0.04
Grade 7 or less	7 (23.33)	10 (33.33)	11 (36.67)	-
Grade 8–Grade 10	17 (56.67)	6 (20.00)	8 (26.67)	-
Grade 11–Grade 12	5 (5.56)	13 (14.44)	10 (33.33)	-
Tertiary	1 (1.11)	1 (1.11)	1 (1.11)	-
**Annual Income**				0.25
≥ 120 000	1 (3.33)	0 (0.00)	0 (0.00)	-
80 000 ≤ 120 000	0 (0.00)	1 (3.33)	3 (10.00)	-
50 000 ≤ 80 000	1 (3.33)	0 (0.00)	0 (0.00)	-
30 000 ≤ 50 000	4 (13.33)	3 (10.00)	0 (0.00)	-
20 000 ≤ 30 000	1 (3.33)	2 (6.67)	4 (13.33)	-
15 000 ≤ 20 000	2 (6.67)	4 (13.33)	6 (20.00)	-
≤ 15 000	1 (3.33)	4 (13.33)	1 (3.33)	-
Unemployed/Pensioner	20 (66.67)	16 (53.33)	16 (53.33)	-

*Source*: Data collected during study

EG1, Experimental group one; EG2, Experimental group two.

**TABLE 5:** Cardiovascular risk factors (*n* = 90; 30 participants per group).

Cardiovascular risk factors	Groups at baseline			*p*-value
		
	EG 1 *n*(%)	EG 2 *n*(%)	CG 3 *n*(%)	
Stroke	3 (10.00)	5 (16.67)	3 (10.00)	0.78
Heart problems	7 (23.33)	9 (30.00)	10 (33.33)	0.77
Eye problems	16 (53.33)	20 (66.67)	17 (56.67)	0.64
Diabetes	5 (16.67)	6 (20.00)	4 (13.33)	0.94
Renal problems	3 (10.00)	1 (3.33)	3 (10.00)	0.69
High cholesterol	8 (26.67)	8 (26.67)	7 (23.33)	1
Smoking	4 (13.33)	2 (6.67)	5 (16.67)	0.61
Drinking alcohol	6 (20.00)	5 (16.67)	5 (16.67)	1
Not Exercising	23 (76.67)	23 (76.67)	26 (86.67)	0.95

EG1, Experimental group one; EG2, Experimental group two; CG3, Control group three.

## Trustworthiness

All the measurements that were used were standardised universally-accepted measures. The patients were given information that was prepared carefully so as to ensure that it was at an appropriate educational level. The exercise programme consisted of walking only, which was within all participants’ capabilities.

### Reliability

All measurements were taken by the first author who had undergone a period of familiarisation and intra-rater reliability checking.

### Validity

All measures are used routinely in studies of this nature and are valid.

## Discussion

The added intervention of the diary appeared to have had no direct effect on BP change greater than that achieved by the introduction of appropriate medication. All groups had similar drops in BP, probably brought about by the medication, with no significant between group differences. All three groups showed a clinically-significant drop in both systolic and diastolic BP that brought their resting values to within accepted norms. The drops were to below the internationally-accepted definition of hypertension values (BP 140/90 mmHg) and systolic BPs dropped by the recommended 4 mmHg – 9 mmHg (physical activity) and 2 mmHg – 8 mmHg (dietary sodium reductions) in all groups except EG2 at six months, which only dropped 2.11 mmHg (SD 23.71).^8^ As this was a group of patients new to the clinic it was felt that the use of a diary that included supporting information should have encouraged patients to adhere better to lifestyle modifications, but this did not seem to have had a beneficial effect on this group of patients^4^,^5^ as tested over this six-month period. It is possible that a longer follow-up period with the diary and its advisory tips and daily reminders might show an improved adherence to lifestyle modification over a longer period.

Previous studies show that it is very difficult to control BP in hypertensive patients.^25, 26^ In a similar way, a study at the same clinic that had a structured home programme of exercise and a once-a-month telephone call to the patients and a member of their family also found no significant changes in BP, but the patients were able to modify some cardiovascular risk factors in that specific supported environment.^7^ However, the participants in that study were not new to the clinic as were the participants in this study. New patients who have not undergone previous treatment in a chronic hypertension unit would be expected to respond better to cardiovascular risk modification treatment than those patients who have been attending the unit for a long period of time, as they might possibly be more receptive to advice on adherence to medication and lifestyle changes.^7^ Although there were no significant between-group differences in the changes in BP, these results suggest that the intervention in EG1 might have been effective in the first six months as this is when the greatest drop in BP occurred. When the telephone reminders were withdrawn after six months, the BP reductions slowed down, suggesting that the diary supported by the telephone reminders might have had a positive effect. The intervention may show a trend of sustained improvement but longer follow up would be necessary in order to evidence this.

Studies have been consistent in showing that despite implementing lifestyle modifications, it is very difficult to reduce weight and BMI in patients with hypertension and to maintain these changes.^7, 27, 28, 29^ The waist–hip ratio in EG1 was found to be marginally significantly reduced compared with the other groups. Waist–hip ratios give an indication of central or abdominal obesity and have been shown to be better indicators of cardiovascular disease resulting from obesity than BMI.^30^ These results indicate that the combination of the diary and telephone calls in EG1 may have influenced the waist–hip ratios in a positive manner and therefore may help in reducing some cardiovascular risk factors in participants such as these. However, despite appropriate dietary recommendations, appropriate food remains inaccessible and unaffordable for poor people, such as the sample in this study, in developing countries, of which South Africa is one.^11^ Healthy food should become accessible and attainable to all in order to help prevent and control chronic lifestyle diseases.^8^

Although there were improvements in exercise tolerance in the experimental groups there was not a significant between-group difference. This may suggest that the telephone calls and diaries were helpful in promoting regular exercise. However, in comparison with the previous study in this clinic, the distances improved considerably less in this study.^7^ Other studies have also shown that home-based exercise programmes improve exercise capacity in patients,^31^ and self-kept diaries of physical activities^18^ and telephone calls^32^ improve adherence to exercise. It is possible that if the study patients had been encouraged more during the six-minute walk test, they may have walked further. This is despite having been encouraged to walk as fast as possible at the start of the test. However, the standard protocol of only one encouraging statement per lap was followed^20^ and it is possible that this statement needs to have been even more encouraging.

Mean glucose dropped to normal levels in all three groups, but then glucose levels were not exceptionally high at baseline. Although not significantly different the two experimental groups showed a decrease in cholesterol and the CG showed an increase in cholesterol over the first six months. As with the glucose results, the greatest improvement in cholesterol was noted in EG1. Miller et al. also showed a drop in cholesterol due to comprehensive lifestyle intervention, although the cholesterol means were much higher in the study by Miller et al. as compared with this study.^3^

Although not significant, the improvement in HDL in EG1 shows that the diary intervention may be effective in raising HDL. This might be due to the daily reminders to exercise. This increase was sustained at nine months. The significant decrease in LDL in both intervention groups at nine months also may be a result of either the diary and/or the telephone calls, results that are similar to studies in the literature.^33^,^34^ Adherence to lifestyle modification is very difficult to achieve and studies have shown this consistently,^32^ especially in poor socio-economic communities.^7, 15, 33, 34, 35^ A far more concerted effort at all levels of health care is needed in order to reduce the current levels of lifestyle diseases. These interventions probably need to begin with children in order to ingrain the good lifestyle choices that promote good health.

## Conclusion

There were no significant differences in the change in BP as a result of the reminder diary and the telephone call. However, the small drop in BP, combined with the improved blood profile, waist–hip ratios and distances walked, provide encouraging findings for the use of a reminder diary plus telephone calls to improve adherence to lifestyle modifications in order to reduce BP. Studies with longer follow up periods may be required in order to establish whether the intervention has long-term effects.

## References

[1] Jokisalo E, Kumpusalo E, Enlund H, et al Factors related to non-compliance with antihypertensive drug therapy. J Hum Hypertens. 2002;16(8):577–583. http://dx.doi.org/10.1038/sj.jhh.1001448, PMid:12149664 12149664 10.1038/sj.jhh.1001448

[2] Gohar F, Greenfield S, Beevers DG, et al Self-care and adherence to medication: a survey in the hypertension outpatient clinic. BMC Complem Altern M. 2008;8(4). 10.1186/1472-6882-8-4 PMC2259297 18261219

[3] Miller ER 3rd, Erlinger TP, Young DR, et al Results of the diet, exercise, and weight loss intervention trial (DEW-IT). Hypertension. 2002;40(5):612–618. http://dx.doi.org/10.1161/01.HYP.0000037217.96002.8E 12411452 10.1161/01.hyp.0000037217.96002.8e

[4] Daniels A, Biesma R, Otten J, et al Ambivalence of primary health care professionals towards the South African guidelines for hypertension and diabetes. S Afr Med J. 2000; 90(12):1206–1211. PMid:11234651 11234651

[5] Hyman DJ, Pavlik VN Characteristics of patients with uncontrolled hypertension in the United States. N Engl J Med. 2001;345(7):479–486. http://dx.doi.org/10.1056/NEJMoa010273, PMid:11519501 11519501 10.1056/NEJMoa010273

[6] Southern African Hypertension Society Executive Committee 2000 Hypertension clinical guideline. S Afr Med J 2000;91(2 Pt 2):163–172. 11288383

[7] Stewart A, Noakes T, Eales C, et al Adherence to cardiovascular risk factor modification in patients with hypertension. Cardiovasc J S Afr. 2005;16(2): 102–107. PMid:15915277 15915277

[8] Seedat YK, Croasdale MA, Milne FJ, et al South African hypertension guideline 2006. S Afr Med J. 2006;96(4 Pt 2):337–362. PMid:16670808 16670808

[9] Katz I, Schneider H, Shezi Z, et al Managing type 2 diabetes in Soweto – the South African chronic disease outreach programme experience. Prim Care Diabetes. 2009; 3(3):157–164. http://dx.doi.org/10.1016/j.pcd.2009.06.007, PMid:19640820 19640820 10.1016/j.pcd.2009.06.007

[10] Steyn K, Bradshaw D, Norman R, et al Determinants and treatment of hypertension in South Africans: The first Demographic and Health Survey. S Afr Med J. 2008; 98(5):376–380. PMid:18637309 18637309

[11] Seedat YK Impact of poverty on hypertension and cardiovascular disease in sub-Saharan Africa. Cardiovasc J Afr. 2007; 18(5):316–320. PMid:17957321 17957321 PMC3975541

[12] Serour M, Alqhenaei H, Al-Saqabi S, et al Cultural factors and patients’ adherence to lifestyle measures. Br J Gen Pract. 2007;57(537):291–295. PMid:17394732, PMCid:2043336 17394732 PMC2043336

[13] Munro S, Lewin S, Swart T, et al A review of health behaviour theories: how useful are these for developing interventions to promote long-term medication adherence for TB and HIV/AIDS? BMC Public Health. 2007;7:(104). PMid:17561997, PMCid:1925084 10.1186/1471-2458-7-104 PMC1925084 17561997

[14] Atreja A, Bellam N, Levy SR Strategies to Enhance Patient Adherence: Making it Simple. MedGenMed; 2005;7(1):4 PMid:16369309, PMCid:1681370 16369309 PMC1681370

[15] Haynes RB, McDonald HP, Garg AX Helping Patients Follow Prescribed Treatment Clinical Applications. JAMA. 2002;288(22):2880–2883. http://dx.doi.org/10.1001/jama.288.22.2880, PMid:12472330 12472330 10.1001/jama.288.22.2880

[16] van Berge Henegouwen MT, van Driel HF, Kasteleijn-Nolst Trenité DG A patient diary as a tool to improve medicine compliance. Pharm World Sci. 1999;21(1): 21–24. 10214664 10.1023/a:1008627824731

[17] Verbrugge LM Health diaries. Med Care. 1980;18(1):73–95. http://dx.doi.org/10.1023/A:1008627824731, PMid:10214664 6986517 10.1097/00005650-198001000-00006

[18] Arrigo I, Brunner-LaRocca H, Lefkovits M, et al Comparative outcome one year after formal cardiac rehabilitation: the effects of a randomized intervention to improve exercise adherence. Eur J Cardiovasc Prev Rehabil. 2008;15(3):306–311. http://dx.doi.org/10.1097/HJR.0b013e3282f40e01,PMid:18525385 18525385 10.1097/HJR.0b013e3282f40e01

[19] Borg GAV Psychophysical basis of perceived exertion. Med Sci Sports Exerc. 1982;14(5):377–381. http://dx.doi.org/10.1249/00005768-198205000-00012 PMid:7154893 7154893

[20] Guyatt GH, Sullivan MJ, Thompson PJ, et al The six minute walk: A new measure of exercise capacity in patients with chronic heart failure. Can Med Assoc J. 1985; 132:919–923. PMid:3978515, PMCid:1345899 3978515 PMC1345899

[21] American College of Sports Medicine Guidelines for exercise testing and prescription. 4th ed Philadelphia, USA: Lea and Febiger; 1991.

[22] Visscher TLS, Seidell JC, Molarius A, et al A comparison of body mass index, waist–hip ratio and waist circumference as predictors of all-cause mortality among the elderly: the Rotterdam study. Int J Obes. Internat; 2001;25(11): 1730–1735. http://dx.doi.org/10.1038/sj.ijo.0801787, PMid:11753597 10.1038/sj.ijo.0801787 11753597

[23] Hanssen TA, Nordrehaug JE, Eide GE, et al Improving outcome after myocardial infarction: a randomized controlled trial evaluating effects of a telephone follow-up intervention. Eur J Cardiovasc Prev Rehabil. 2007;14(3):429–437. http://dx.doi.org/10.1097/HJR.0b013e32801da123, PMid:17568244 17568244 10.1097/HJR.0b013e32801da123

[24] Gauteng Department of Health Guide to a healthy eating plan. South Africa: Government Printers; 2008.

[25] Rayner B, Schoeman HS A cross-sectional study of blood pressure control in hypertensive patients in general practice (the I-TARGET study). Cardiovasc J Afr. 2009;20(4):224–227. PMid:19701531 19701531 PMC3721771

[26] Phillips L, Branch W, Cook C, et al Clinical inertia. Ann Intern Med. 2001;135 (9):825–834. http://dx.doi.org/10.7326/0003-4819-135-9-200111060-00012, PMid:11694107 11694107 10.7326/0003-4819-135-9-200111060-00012

[27] Burke LE, Swigart V, Turk MW, et al Experiences of self-monitoring: successes and struggles during treatment for weight loss. Qual Health Res. 2009;19(6):815–828. http://dx.doi.org/10.1177/1049732309335395, PMid:19365099, PMCid:2855299 19365099 10.1177/1049732309335395 PMC2855299

[28] Shay L, Seibert D, Watts D, et al Adherence and weight loss outcomes associated with food-exercise diary preference in a military weight management programme. Eat Behav. 2009;10(4):220–227. http://dx.doi.org/10.1016/j.eatbeh.2009.07.004, PMid:19778751 19778751 10.1016/j.eatbeh.2009.07.004 PMC3936599

[29] Beasley JM, Riley WT, Davis A, et al Evaluation of a PDA-based dietary assessment and intervention programme: A randomized controlled trial. J Am Coll Nutr. 2009;27(2):280–286. 18689560 10.1080/07315724.2008.10719701

[30] Yusuf S, Hawken S, Ounpuu S, et al: INTERHEART Study Investigators. Obesity and the risk of myocardial infarction in 27,000 participants from 52 countries: a case-control study. Lancet. 2005; 366(9497):1640–1649. http://dx.doi.org/10.1016/S0140-6736(05)67663-5 16271645 10.1016/S0140-6736(05)67663-5

[31] Tully MA, Cupples ME, Chan WS, et al Brisk walking, fitness and cardiovascular risk: A randomized controlled trial in primary care. Prev Med. 2005;41(2):622–628. http://dx.doi.org/10.1016/j.ypmed.2004.11.030, PMid:15917061 15917061 10.1016/j.ypmed.2004.11.030

[32] Sacks FM, Svetkey LP, Vollmer WM, et al Effects on blood pressure of reduced dietary sodium and the dietary approaches to stop hypertension (DASH) diet. N Engl J Med. 2001;344(1):3–10. http://dx.doi.org/10.1056/NEJM200101043440101, PMid:11136953 11136953 10.1056/NEJM200101043440101

[33] Brunenberg D, Wetzels G, Nelemans P, et al Cost effectiveness of an adherence-improving programme in hypertensive patients. Pharmacoeconomics. 2007;25(3): 239–251. http://dx.doi.org/10.2165/00019053-200725030-00006, PMid:17335309 17335309 10.2165/00019053-200725030-00006

[34] Steyn K, Gaziano TA, Bradshaw D, et al Hypertension in South African adults: results from the Demographic and Health Survey, 1998. J Hypertens. 2001;19(10):1717–1725. http://dx.doi.org/10.1097/00004872-200110000-00004, PMid:11593090 11593090 10.1097/00004872-200110000-00004

[35] Seedat YK Perspectives on research in hypertension. Cardiovasc J Afr. 2009;20(1): 39–42. PMid:19287815 19287815 PMC4200561

